# Ankaferd hemostat: from molecules to medicine

**DOI:** 10.3906/sag-1908-161

**Published:** 2020-11-03

**Authors:** Rafiye ÇİFTÇİLER, İbrahim Celalettin HAZNEDAROĞLU

**Affiliations:** 1 Department of Hematology, Faculty of Medicine, Hacettepe University, Ankara Turkey

**Keywords:** Ankaferd hemostat, proteomic analyses, molecules, transcription factors

## Abstract

Ankaferd hemostat (ABS; Ankaferd Blood Stopper®, İstanbul, Turkey) is a hemostatic agent having an impact on red blood cell–fibrinogen interactions. The hemostatic effect of ABS depends upon the quick promotion of a protein network, particularly fibrinogen gamma, in relation to the erythrocyte aggregation. The entire physiological process involves ABS-induced formation of the protein network by vital erythrocyte aggregation. Vital erythrocyte aggregation occurs with the spectrine, ankyrin, and actin proteins on the membrane of the red blood cells. ABS notably affects cell metabolism and cell cycle mechanisms. Meanwhile, ABS has antiproliferative effects on cancer cells. The aim of this review is to assess molecular basis of ABS as a hemostatic drug. The literature search on ABS was performed in PubMed, Web of Science (SCI expanded), and Scopus with particular focus on the studies of molecular basis of ABS, in vivo research, case series, and controlled randomized clinical studies. Current perspective for the utilization of ABS is to provide hemostasis with accelerating wound healing. Future controlled trials are needed to elucidate the pleiotropic clinical effects of ABS such as antineoplastic, antiinflammatory, antiinfective, antifungal, and antioxidative effects.

## 1. Introduction

Ankaferd hemostat (ABS; Ankaferd Blood Stopper®, İstanbul, Turkey) had been traditionally practiced in Anatolia as a hemostatic agent for centuries. It is a hemostatic agent having an impact on the red blood cell–fibrinogen interactions [1]. ABS contains a standardized combination of the plants named as *Glycyrrhiza glabra* , *Thymus vulgaris* , *Alpinia ofﬁcinarum* , *Vitis vinifera* , and *Urtica dioica* [2,3]. ABS shows its hemostatic effect via affecting the physiology of red blood cells [1]. ABS has an expanding spectrum of clinical indications. There are distinct and important molecular components of the ABS-induced hemostatic network. Vital erythrocyte aggregation is established with spectrin, ankyrin, and actin proteins on the membranes of red blood cells. Recently, ABS has been introduced as a new topical hemostatic agent when conventional methods for the management of clinical bleeding are not effective [4–6]. ABS is also effective in patients with primary or secondary hemostatic dysfunction as well as in patients with normal hemostatic parameters and bleeding [7,8]. The aim of this review is to assess essential molecular basis of ABS as a hemostatic drug.

## 2. Proteomic analyses of ABS

Demiralp et al. disclosed the protein library of ABS via MALDI-TOF analysis. The whole physiological activity involves ABS-induced formation of the protein network by vital erythrocyte aggregation. Vital erythrocyte aggregation occurs with the spectrin, ankyrin, and actin proteins on the membrane of the red blood cells. Mandatory erythroid proteins (ankyrin recurrent and FYVE bundle containing protein 1, spectrin alpha, actin-depolymerization factor, actin-depolymerizing factor, LIM bundle and actin binding subunit 1 isoform a, LIM bundle and actin binding subunit 1 isoform b, NADP-dependent malic enzyme, NADH dehydrogenase (ubiquinone) 1 alpha subcomplex, mitochondrial NADP (+)-dependent malic enzyme 3, ribulose bisphosphate- carboxylase large chain, maturase K) and the necessary ATP bioenergy (ATP synthase, ATP synthase beta subunit, ATP synthase alpha subunit, ATP-binding protein C12, TP synthase H+ transporter protein, ADF, and alpha-1, 2 glycosyltransferase ALG10-A) are the main components of protein library of ABS [9]. ABS also regulates GATA/FOG transcription system affecting erythroid functions and urotensin II [9,10]. Urotensin II, a very important component of ABS, also represents the relationship between injured vascular endothelium, adhesive proteins, and active erythroid cells [9].

**Figure 1:**
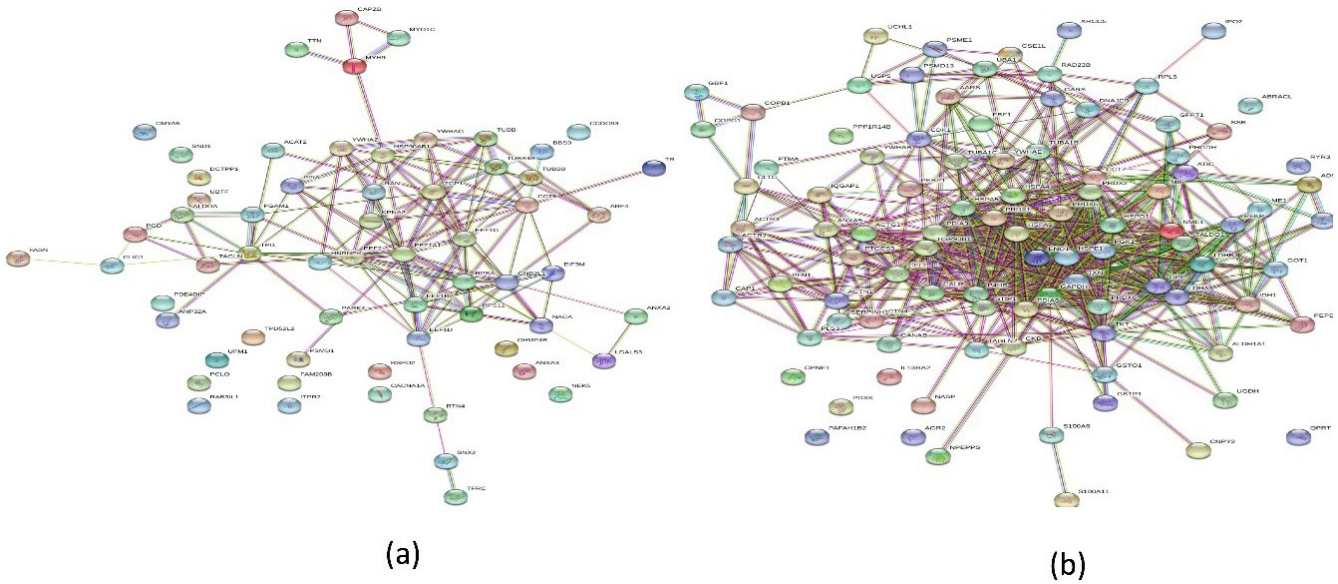
(a) The Map of down-regulated and (b) the Map of upregulated proteins with the administration of Ankaferd hemostat (ABS) upon human colon cancer cell line, CACO-2. (reproduced with the open access policy of the Journal; Bio Med Res (https://www.hindawi.com/journals/bmri/2019/5268031/fig4/))

**Figure 2:**
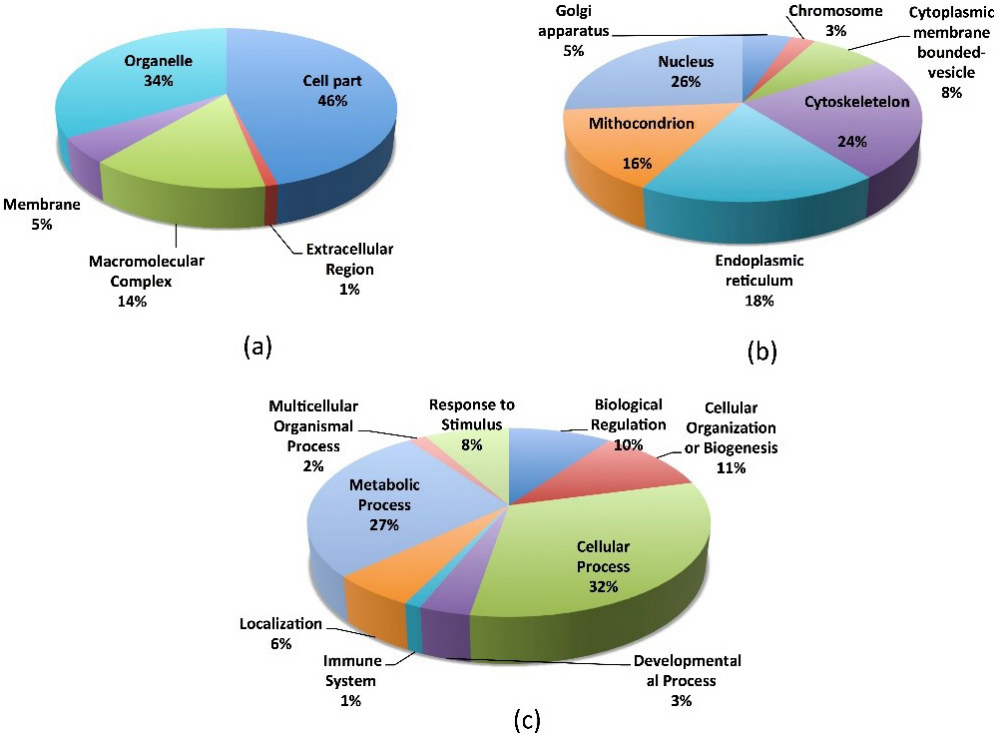
(a) The classification of upregulated proteins in cellular component. (b) Classification of upregulated proteins in organelles. (c) Classification of upregulated proteins in cellular processes affected by the administration of Ankaferd hemostat (ABS) in the pathobiology of colon cancer cell line (https://www.hindawi.com/journals/bmri/2019/5268031/fig3/).

ABS particularly affects cell metabolism and cell cycle mechanism and has antiproliferative effects on cancer cells. ABS affects the potential targets on cancer treatment such as SND1, KPNA2, and PARK7. Another important outcome was the upregulation of tumor suppressor proteins UCHL1 and RPL5. In particular, RPL5 directly activates the p53 apoptotic pathway and causes apoptosis [11]. Protein–protein interaction networks are important interactions in understanding cellular processes such as metabolism, signal transduction, and drug resistance. The interactions of the identified proteins were mapped as depicted in Figure 1. These proteins were linked and worked together to protect the Caco-2 cells against the effect of ABS as depicted in Figure 2 [11].

## 3. The effects of ABS on transcription factors

The effects of ABS on transcription factors and erythrocyte protein profile in HUVEC endothelium were investigated. In one study, ABS was shown to be highly effective not only outside the cells but also inside the cell at low doses (5 μL) and could affect many molecular mechanisms in endothelial cells. The level of the activity of the transcription factors (AP2, AR, CREATF1, CREB, E2F1-5, E2F6, EGR, GATA, HNF1, ISRE, Myc-Max, NF1, NF-κB, p53, PPAR, SMAD2/3, SP1, TRE/AP1, and YY1) were significantly increased in response to ABS. Those transcription factors regulate a wide variety of biological functions, including hemostasis, infection, cellular proliferation, and inflammation [12]. GATA regulates erythroid differentiation and promotes the production of erythroid proteins such as spectin. In another study, GATA activity was significantly increased after ABS administration [12]. ABS importantly increased the level of activity of the following transcription factors; AP2, AR, CRE-ATF1, CREB, E2F1-5, E2F6, EGR, ISRE, Myc-Max, NF1, NF-κB, p53, PPAR, SMAD2/3, SP1, TRE/AP1, and YY1. Those regulator molecules affect different steps of cellular proliferation, such as cell cycle regulation, signal transduction, angiogenesis, apoptosis, inflammation, acute phase reaction, immunity, and several metabolic molecular pathways [12].

## 4. The effects of ABS on the cellular biology 

The antineoplastic effect of ABS was preliminarily defined in cancer cell lines [13,14]. In a study, a dose-dependent inhibition of cell proliferation and a significant reduction in the survival of SAOS-2 cells were observed after ABS administration [13]. In another study, the antineoplastic effects of ABS on colon cancer cells were also defined. Following the addition of ABS to the culture medium, the inhibition of cellular reproduction, and loss in the viabilities of human colon CaCo-2 cells were observed [14]. Mumcuoglu et al. demonstrated that, depending on the concentration and duration of the application, ABS could cause apoptosis via regulating PAR1 and p53-independent p21 involvement in apoptosis stimulation within leukemia cells [15]. Likewise, Akalın et al. showed that the antiproliferative effects of ABS on lymphoid neoplastic cells (B-CLL and RAJI tumor cell lines). First, the inflation of the hematopoietic tumor cells was observed with the addition of ABS solution. The inflation and proliferation continued on B-CLL cells at day 3 and produced aggregation islands [16]. Turk et al. also demonstrated that the most resistant cell type was SK-MEL-10 and the least resistant neoplastic cell type was A2058. The anticancer effect of ABS was also evident on light microscopic images of untreated M307 primary cells [17]. Another study revealed that ABS induces DNA damage, apoptosis, and cytotoxic activity via generating reactive oxygen species in melanoma cell lines [18]. One study investigated the biological activity of ABS-derived iron on iron-regulated genes during iron-deficiency anemia. The results showed that ABS-derived iron-influenced transcriptions of iron-regulated marker genes, including divalent metal transporter (Dmt1), transferrin receptor (TfR), ankyrin repeat domain 37 (Ankrd37), and hepcidin (Hamp). ABS might have an ability to reduce levels of iron deficiency anemia [19].

## 5. The principal effect of ABS on hemostatic processes

ABS contains essential erythroid proteins such as spectrin-alpha, actin depolymerization factor, NADH dehydrogenase (ubiquinone) 1-alpha subcomplex, mitochondrial NADP (+)-dependent malic enzyme and the required adenosine triphosphate (ATP) bioenergy source [1]. In the presence of ABS, vital erythrocyte aggregation occurs via spectin and ankyrin receptors in red blood cell membranes [20]. Moreover, there are distinct mechanisms affecting hemostatic plug formation. ABS also affects different regulator molecules such as protein-2 (AP2), androgen receptor (AR), cyclic AMP response element or activating transcription factor-1 (CRE-ATF1), cyclic AMP response element binding protein (CREB), E2F1-5, E2F6, interferon (IFN)-stimulated response element (ISRE), Myc-Max, nuclear factor-1 (NF-1), protein-53 or tumor protein-53 (p53), peroxisome proliferator-activated receptor (PPAR), and Yin Yang-1 (YY1) transcription factors. Those regulatory molecules affect cellular vascular hemostasis, angiogenesis, signal transduction, apoptosis, inflammation, acute-phase reaction, immunity and several metabolic pathways [9].

**Figure 3:**
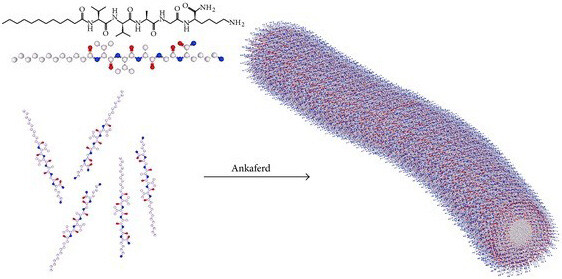
The presentation of ABS Nanohemostat formation by self-assembly of peptide amphiphile molecules into nanofibers upon addition of Ankaferd hemostat solution (https://www.hindawi.com/journals/ijbm/2013/949460/fig2/).

**Figure 4:**
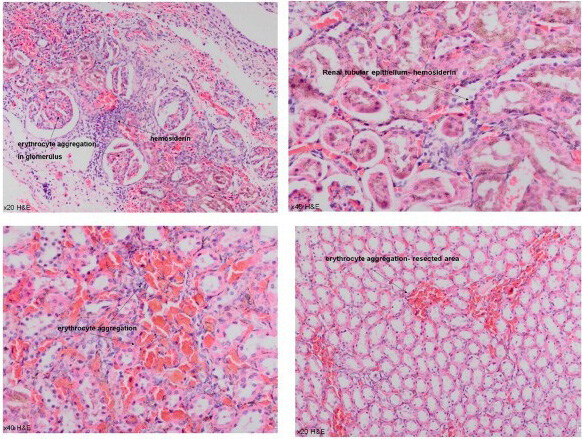
Erythrocyte aggregation in glomerular field and interstitium in ABS Nanohemostat group (20–40xHE) (https://www.hindawi.com/journals/ijbm/2013/949460/fig7/).

Nanotechnological approaches were applied to further develop ABS drug [21,22]. Using the classical ABS solution to create active hemostasis during partial nephrectomy could not be so efficient due to the insufficient contact surface between the ABS hemostatic liquid agent and the bleeding area. In order to expand the contact surface, Huri et al. generated a chimeric hemostatic agent, ABS nanohemostat, via combining a self-assembling peptide amphiphile molecule with the traditional ABS as depicted in Figure 3. ABS nanohemostat has comparable hemostatic efficacy to the traditional ABS in the partial nephrectomy experimental model as shown in Figure 4. Clarification of the cellular and tissue effects of this chimeric compound may open new avenues for novel experimental and clinical studies in the hemostasis [22].

Coagulation proteins (Factors V, VII, VIII, IX, X, XI, and XIII), prothrombin time (PT) and activated partial thromboplastin time (aPTT) were normal during ABS administration. However, it was observed that thrombin time (TT) was prolonged due to fibrinogen gamma [1]. On the contrary, a recent hemorheological study showed that ABS has antierythrocyte aggregation effect. ABS inhibits pathological aggregation of red blood cells. Antithrombotic clinical effects of ABS could be ascribed to the paradoxal antierythrocyte aggregation actions of the drug [23].

## 6. Preclinical in vivo animal models of ABS

Numerous studies have been conducted on preclinical animal models to demonstrate the efficacy and safety of ABS [24–29]. Kuru et al. observed that rectal administration of ABS showed positive effects on bursting pressures, tissue prolidase and hydroxyproline levels and the histopathological findings of colonic anastomosis [30]. The administration of ABS to the superficial and deep abdominal lacerations could control bleeding successfully [24]. In another animal study, the effectiveness of ABS was compared to several conventional antihemorrhagic methods. Hemostasis was successfully achieved via ABS application. Moreover, warm ischemia times decreased by ABS [31]. In another study, the rats underwent femoral vein puncture. One subgroup was treated with ABS tampon or spray and the other control group was left untreated. After two weeks, each group underwent partial tissue excision from the same femoral region, brain, heart, kidney, and liver. ABS was observed to stop the bleeding. There were no histopathological changes at the tissue level and no pathological effects in other organ tissues under light microscope [32]. Beyazit et al. investigated the macroscopic and microscopic changes in the cervical mucosa and immunohistochemical staining for IL-1 β in response to ABS treatment in a chemically induced cervicitis model of rats. The study showed that ABS had significant protective effects on vascular congestion and cervical erosion [33].

## 7. Clinical usage of ABS 

ABS has hemostatic, antithrombotic, antiinfective, antineoplastic, and wound healing effects. ABS has also been employed in controlled clinical trials as depicted in Table.

**Table:** Clinical studies of Ankaferd hemostat.

Authors	Clinical study	Study population	Study design and results
Koyuncu et al.	Advances in therapy 2019;36: 1143-1149 [34]	80 patients with bleeding associated with extremity lacera-tions	ABS appears to be useful in controlling bleeding due to lacerations on the extremities in adults. Bleeding was stopped significantly faster and recurred significantly less frequently in the ABS-treated group.
Gorgulu et al.	J Interven Cardiol. 2018;1-7 [35]	630 patients underwent coronary angiography	It is possible that ABS can completely prevent radial artery occlusion and ABS is effective in preventing bleeding complications.
Yaman et al.	Eur Arch Paediatr Dentist2012; 13: 197-202 [36]	30 patients with dental problems	ABS was compared with formocresol for pain, swelling, mobility, resorption, furcation, and periapical bone destruction. ABS was as effective as formocresol for a pulp dressing of primary molar.
Iynen et al.	Int J Pediatr Otorhinolaryngol2011; 75: 1292-1295 [37]	90 patients who underwent adenoidectomy	Clinical effect of ABS on hemostasis on peri-adenoidectomy period was evaluated. ABS decreases the duration and quantity of blood loss postadenoidectomy and increases postoperative quality of life.
Teker et al.	European Archives of Oto-Rhino-Laryngology, 2010, 267.9: 1377-1381 [38]	49 patients with epistaxis	Hemostasis by ABS was compared to hemostasis by phenylephrine. ABS was an effective, safe, rapid, and simple alternative to the phenylephrine in patients with epistaxis.
Teker et al.	Int J Pediatr Otorhinolaryngol2009; 73: 1742-1745 [39]	47 patients with chronic tonsillitis, tonsillar hypertrophy, and obstructive sleep apnea syndrome	ABS reduced intraoperative hemorrhage and operation time for patients with chronic tonsillitis, tonsillar hypertrophy, and obstructive sleep apnea syndrome
Guler et al.	J Invest Surg 2011; 24:205–210 [40]	61 patients who underwent thyroidectomy	ABS and hemostasis by conventional technique (HCT) groups were compared in terms of operation time, postoperative drainage, duration of postoperative hospitalization and complications. ABS was more effective than HCT to control hemorrhage following total thyroidectomy
Pamuk et al.	J Periodontal Res 2016;51: 540-547 [41]	15 patients with periodontitis	ABS improved soft tissue healing during the periodontal defect filling by the ACB by stimulating angiogenesis and vascular endothelial cell function.
Istanbulluoglu et al.	J Endourol 2013; 27: 1126-1130 [42]	90 patients who underwent percutaneous nephrolithotomy	45 of the patients underwent tubeless percutaneous nephrolithotomy (PCNL) with the use of ABS as a hemostatic agent, whereas the remaining ones underwent tubeless PCNL without ABS. ABS is a potent and safe hemostatic agent in tubeless PCNL.
Amer et al.	Eur J Dent 2014; 8: 475-480[43]	205 patients who underwent dental procedures	Patients were selected so that 80 patients have INR values of ≤2, whereas the remaining patients have the INR values ranging from 2 to 3 and the procedures were applied. ABS is an effective hemostatic agent comparable to tranexamic acid in controlling postextraction bleeding in AOT patients of INR values ≤3.
Atalay et al.	Open Cardiovasc Med J 2015;9: 18-25 [44]	50 patients who underwent CABG surgery	25 CABG patients received a high-dose clopidogrel (600 mg) and 300 mg ASA have been included in the study (ABC group). Twenty-five patients have also been included as control group. Local use of ABC reduced bleeding from the mediastinum after CABG.
Yasar et al.	Afr J Tradit Complem 2011;8: 444-446 [45]	60 patients who underwent adenoidectomy	A significantly shorter duration of bleeding and a lower number of packs are needed to obtain ABS tamponade-induced hemostasis during adenoidectomy as compared to saline soaked gauze sponge application.
Akpinar et al.	Heart Surg Forum 2015; 18: E118–E123 [46]	50 patients who underwent CABG	Twenty-five CABG patients premedicated with clopidogrel and ASA were included and 25 patients who were premedicated with the same antiplatelet agents were as a control group. The use of local ABS decreases bleeding and transfusion requirements in patients premedicated with clopidogrel and ASA undergoing emergent CABG.
Eyi et al.	Clin Exp Obstet Gynecol 2013;40: 141-143 [47]	40 pregnant women who required a mediolateral episiotomy	The patients were randomly assigned to two approaches on ABS and SS. Application of 4 mL of ABS compared to SS lessened bleeding.
Atay et al.	Int J Hematol Oncol 2015; 25: 166–171 [48]	20 patients with oral mucositis	The healing duration of oral mucositis was shorter with the topical ABS application. Additionally hemorrhages from oral mucositis lesions were healed within 2 days with ABS.

ACB: autogenous cortical bone greft, ABS: Ankaferd blood stopper; AOT: Oral anticoagulant therapy; CABG: coronary artery bypass grafting; ASA: acetylsalicylic acid; INR: international normalized ratio; GIS: gastrointestinal system; GR: gingival recession; HCT: hemostasis by conventional technique; PCNL: percutaneous nephrolithotomy; SS: saline solution; VEGF: vascular endothelial growth factor.

### 7.1. The usage of Ankaferd Hemostat (ABS) in bleeding

Clinical studies have demonstrated that ABS can be safely and effectively used in surgical and dental procedures in patients with normal and abnormal hemostasis. Huri et al. reported a case indicating the efficacy of ABS in a patient with prostate adenocarcinoma undergoing radical retropubic prostatectomy. Hemostasis was achieved with ABS in active bleeding tissues during radical prostatectomy [49]. In another study, ABS was successfully applied to control bleeding without suturing the renal parenchyma. ABS was shown to have a significant effect on active hemostasis during urogenital operations [50].

Gastrointestinal (GI) bleeding is an important life-threatening condition and a common cause of hospitalization. During the research for a further hemostatic agent for the management of GI hemorrhages, current evidence suggests that ABS could have an efficient hemostatic effect for the “difficult-to-manage” subtypes of GI hemorrhages [51–53]. Ozaslan et al. showed that five adult patients with bleeding peptic ulcer disease, in which ABS was used as a primary hemostatic agent, were reported to achieve success in control of bleeding within minutes [54]. Additionally, Purnak et al. reported a successful hemostasis in a patient with a bleeding peptic ulcer complicated with defective hemostasis [55]. There are also numerous case reports showing that ABS was used safely in GI bleeding in infants and children. The pediatric experience with ABS in an infant with bleeding peptic ulcer had been recently demonstrated. ABS was applied over the ulcer surface and in a very short period white-grey adherent clot was developed on the ulcer and bleeding ceased [56]. 

Yasar et al. evaluated the efficacy of ABS tamponade in the control of intra-operative bleeding occurring during adenoidectomy performed in children under the age of 12 [45]. In all countries, cardiac surgeries are increasing and parallel to this, sternotomy healing and bleeding problems cause surgeons to search for new options and use several new techniques and materials. Ergeneoglu et al. showed that applying ABS was a useful different technique for the control of sternal bleeding during cardiac surgery [57]. Another study showed that the local use of ABS decreases bleeding significantly during the operation. Therefore, transfusion requirements of erythrocyte suspension and platelets decreased in patients who received clopidogrel and ASA undergoing emergent operations [44]. 

### 7.2. The usage of ABS in wound healing

Functional proteomic analyses had defined antithrombin and prohemostatic activities of ABS which are related to fibrinogen gamma chain and prothrombin [58]. ABS can improve the wound-healing process via providing inhibition of extra cellular matrix-degrading enzymes during wound repair. Moreover, ABS enhanced the stimulated migration of 3T3 fibroblasts to an artificial wounded area [59]. Plant extracts in ABS have been reported to inhibit various enzymes. The extracts of *T. vulgaris* were shown to inhibit collagenase and elastase inhibition by 25% and 17%, respectively, while hyaluronidase was shown to be inhibited 100% [60]. Additionally, another study showed that ursolic acid extracted from *U. dioica* exhibited elastase and collagenase inhibition activity of 24.5% and 16.2%, respectively [61]. Topal et al. showed that the wound contraction in the ABS and silver sulphadiazine groups was significantly higher than in the control group on days 14, 21, and 28 and suggested that ABS could be successfully used for burn wound healing besides silver sulphadiazine [62]. ABS may be useful in the treatment of acute burn lesions. It was observed that the wounds healed rapidly with the topical application of ABS onto the burn lesions [63]. In one study, the effect of ABS on the cut surface of the pancreatic duct as well as the pancreatic remnant was a safe procedure that prevents the formation of pancreatic fistula without causing adverse side effects such as pancreatitis [64]. Radiation-induced esophageal ulcer in a patient with breast cancer was completely healed with topical application [65]. It has been shown that ABS has a protective effect against intestinal damage in an experimental rat necrotizing enterocolitis model due to its antioxidant, antiinflammatory, and antiapoptotic characteristics [66]. In another study, intravaginal ABS injection caused a significant decrease in the number of inflammatory cells in the cervical mucosa and ABS had significant protective effects on cervical erosion [33]. In another animal study, it was observed that ABS administration improved uterine fibrosis and histopathological inflammation by lowering the level of inflammatory markers such as IL-1 and IL-6 [67].

### 7.3. The usage of ABS in infections

*Vitis vinifera* , one of the components of ABS has potential prebiotic effects on modulating the gut microbiota composition and generating SCFAs that contribute to the improvements of host health [68]. ABS was evaluated on 102 clinical isolates from both gram-negative and gram-positive bacteria and four standard strains, including *MRSA ATCC 43300, MSSA ATCC 25923, P. aeruginosa ATCC 27853* and *E. coli ATCC 35218.* The study showed that ABS was significantly active against all of the bacteria investigated [69]. Another study disclosed that ABS is highly effective against several gram-negative and gram-positive bacteria including frequent foodborne microorganisms [70]. Akkoc et al., using agar well diffusion test, found that ABS has a high antifungal effect against *Zygosaccharomyces bailii* , *C albicans* , *Aspergillus flavus* , and *Aspergillus parasiticus* [71]. In another study, ABS reported that when applied directly to *Candida* species, there were changes in growth conditions [72]. The efficiency of ABS on *H. pylori* was also demonstrated. Possible anti- *H. pylori* effect of ABS will broaden the therapeutic spectrum of the drug in GI lesions as peptic ulcer disease (PUD) and lymphoid tissue (MALT) lymphomagenesis [73]. In vitro activity of ABS against *M. Tuberculosis* isolates was evaluated. It was concluded that ABS solution used topically was active against *tuberculosis* bacilli in vitro. Therefore, ABS might be used as a supportive agent in combination with anti- *tuberculosis* drugs during debridement of multidrug resistant *M. tuberculosis* osteomyelitis and lymphadenitis [74]. In one study, the efficacy of ABS with hydatid cysts was compared with other antiseptic agents. It can be said that ABS is an effective antiseptic agent for percutaneous interventions to treat hydatid cysts [75]. In a study, the efficacy of ABS in prophylaxis and oral mucositis treatment in patients receiving chemotherapy in childhood was evaluated. ABS was thought to be effective in prophylaxis and treatment of oral mucositis secondary to chemotherapy in childhood cancers [76].

### 7.4. Toxicity profile of ABS

Quantitative analysis of heavy metals in the ABS sample revealed that there were no Pb, Cd, Hg, and As in ABS. Chromatographic analysis of pesticide analysis in ABS sample revealed that ABS does not contain pesticides. In the analysis of mycotoxin detection using the HPLC method in the ABS drug, ABS was revealed not to contain mycotoxins (i.e. Aflatoxin B1, Aflatoxin B2, Aflatoxin G1, and Aflatoxin G2; total aflatoxins are not present inside ABS). GMO analyses of the detection of genetically altered organisms in the ABS sample represented that no plasmid extraction was performed and ABS did not have GMO treatment during the preparation process. Dioxin analyses in the ABS sample showed that ABS does not contain toxic dioxin and dioxin-like chemical compounds. Thus, the available toxicological results have shown the safety of ABS [77].

### 7.5. Intratumoral ABS for controlling the growth of solid tumors and hepatic metastases

In a previous study, the antineoplastic effects of ABS on myeloma cell line, in vitro and plasmocytoma development in Balb/c mice were investigated by intraperitoneal preterm injection in vitro. The cytotoxicity of ABS against the MM cell lines was determined by the 3-(4,5-dimethylthiazol-2-yl)-2,5-diphenyltetrazolium bromide-dye reduction assay [78]. It was shown that chemopreventive effects of ABS in 7,12-dimethylbenz[a]anthracene (DMBA) induced oral epithelial dysplasia. Histological studies have shown that the buccal pouches of animals treated with DMBA alone showed up serious dysplasia while only moderate or no dysplasia were noticed in DMBA+ABS group [79]. ABS has been used as an embolizing agent in renal and splenic arteries by Medical Interventional Radiology for medical nephrectomy and splenectomy purposes [80,81]. Under fluoroscopic guidance, 2 mL of ABS mixed with 2 mL of nonionic contrast agent (Iopromide) was slowly injected until a resistance to injection secondary to embolization was observed. Slow flow and stagnation were observed with fluoroscopy. Approximately 3.5–4 mL of the mixture was used for embolization [80,81]. In one study, ABS was used instead of alcohol for hepatic embolization in mice. There was a statistically significant difference in the development of necrosis in ABS-treated rats compared to ethanol and saline group [82]. ABS has antineoplastic effects in colon cancer [11]. In colon cancer liver metastases and hepatocellular cancer patients, tumor ablation treatment could be applied via interventional radiology techniques in future clinical practice of ABS. In the current technique: alcohol, N-butyl-2-cyanoacrylate, and caustic substances are used as tumor embolizers. In clinical practice in colon cancer liver metastases and hepatocellular cancer patients, ABS could be used for the palliative, adjuvant, neo-adjuvant, or supportive use via interventional radiology techniques for the management of solid tumors. This hypothesis should be tested in future studies.

## 8. Conclusion

ABS acts as a hemostatic agent in various clinical hemorrhages and has many pleiotropic effects. The hemostatic effect of ABS depends upon the quick promotion of a protein network, especially fibrinogen gamma, in relation to the erythrocyte aggregation. The entire physiological process involves ABS-induced formation of the protein network by vital erythrocyte aggregation. Vital erythrocyte aggregation occurs with the spectrine, ankyrin, and actin proteins on the membrane of the red blood cells. ABS notably affects cell metabolism and cell cycle mechanism. ABS has antiproliferative effects on cancer cells. The expanding spectrum of ABS includes antiinfective, antineoplastic, and wound-healing properties. Current perspective for using ABS is to provide hemostasis and accelerating wound healing. Future controlled trials are needed to show the pleiotropic effects of ABS such as antineoplastic, antithrombotic, antiinflammatory, antiinfective, antifungal, and antioxidative effects.

## References

[ref1] (2010). Evaluation of hemostatic effects of Ankaferd as an alternative medicine. Alternative Medicine Review.

[ref2] (2008). Haemostatic actions of the folkloric medicinal plant extract Ankaferd Blood Stopper®. Journal of International Medical Research.

[ref3] (2005). Identification of volatile components in basil (Ocimum basilicum L.) and thyme leaves (Thymus vulgaris L.) and their antioxidant properties. Food Chemistry.

[ref4] (2008). Haemostatic actions of the folkloric medicinal plant extract Ankaferd Blood Stopper. Journal of International Medical Research.

[ref5] (2010). The use of Ankaferd blood stopper in a patient with Glanzmann’s thrombasthenia with gingival bleeding. Blood Coagulation & Fibrinolysis.

[ref6] (2009). Haemostatic actions of the folkloric medicinal plant extract AnkaferdBlood Stopper. The Journal of international medical research.

[ref7] (2009). Molecular basis of the pleiotropic effects of Ankaferd Blood Stopper. International Union of Biochemistry and Moleculer Biology Life.

[ref8] (2010). Safety and efficacy of Ankaferd Blood Stopper in dental surgery. International Journal of Hematology and Oncology.

[ref9] (2010). Functional proteomic analysis of Ankaferd Blood Stopper. Turkish Journal of Hematology.

[ref10] (2009). Use of a new local haemostatic agent Ankaferd blood stopper after surgical excision of eruption cyst: a case report. International Journal of Oral and Maxillofacial Surgery.

[ref11] (2019). Analysis of the Antiproliferative Effect of Ankaferd Hemostat on Caco-2 Colon Cancer Cells via LC/MS Shotgun Proteomics Approach.

[ref12] (2011). The effects of Ankaferd Blood Stopper on transcription factors in HUVEC and the erythrocyte protein profile. Turkish Journal of Hematology.

[ref13] (2008). Anti-cancer activity of ankaferd blood stopper on osteosarcom (SAOS-2) cell lines in vitro. Istanbul, Naviga Publications.

[ref14] (2008). Anti-cancer activity of Ankaferd on human colon cancer (CACO-2) in vitro. Istanbul, Naviga Publications.

[ref15] (2015). Stopper induces apoptosis and regulates PAR1 and EPCR expression in human leukemia cells. Egyptian Journal of Medical Human Genetics.

[ref16] (2014). Acute in vitro effects of ABS (Ankaferd Hemostat) on the lymphoid neoplastic cells (B-CLL and RAJI tumor cell lines). International Journal of Hematology and Oncology.

[ref17] (2017). Growth inhibitory activity of Ankaferd hemostat on primary melanoma cells and cell lines. SAGE open medicine.

[ref18] (2017). Ankaferd hemostat induces DNA damage, apoptosis and cytotoxic activity by generating reactive oxygen species in melanoma and normal cell lines. International Journal of Clinical and Experimental Medicine.

[ref19] (2018). Ankaferd influences mRNA expression of iron-regulated genes during iron-deficiency anemia. Clinical and Applied Thrombosis/Hemostasis.

[ref20] (2012). Pleiotropic cellular, hemostatic, and biological actions of Ankaferd hemostat. Critical reviews in oncology/hematology.

[ref21] (2016). Ultrastructural analyses of the novel chimeric hemostatic agent generated via nanotechnology, ABS nanohemostat, at the renal tissue level. SpringerPlus.

[ref22] (2013). Generation of chimeric “ABS Nanohemostat” complex and comparing its histomorphological in vivo effects to the traditional Ankaferd hemostat in controlled experimental partial nephrectomy model. International journal of biomaterials.

[ref23] (2019). Effects of Ankaferd Hemostat on red blood cell aggregation: a hemorheological study. Turkish journal of medical sciences.

[ref24] (2009). Hemostatic efficacy of Ankaferd Blood Stopper® in a swine bleeding model. Medical Principles and Practice.

[ref25] (2010). Demonstration of the histopathological and immunohistochemical effects of a novel hemostatic agent, Ankaferd Blood Stopper, on vascular tissue in a rat aortic bleeding model. Journal of cardiothoracic surgery.

[ref26] (2010). Haemostatic role and histopathological effects of a new haemostatic agent in a rat bladder haemorrhage model: an experimental trial. BJU international.

[ref27] (2009). Evaluation of a new hemostatic agent Ankaferd Blood Stopper in experimental liver laceration. Journal of Investigative Surgery.

[ref28] (2009). The efficacy of Ankaferd Blood Stopper in antithrombotic drug-induced primary and secondary hemostatic abnormalities of a rat-bleeding model. Blood Coagulation & Fibrinolysis.

[ref29] (2010). Protective value of a folkloric medicinal plant extract against mortality and hemorrhage in a life-threatening renal trauma model. Urology.

[ref30] (2017). Does the application of Ankaferd Blood Stopper rectally have positive effects on the healing of colorectal anastomosis and prevention of anastomotic leakage? An experimental study. Biomedicine & Pharmacotherapy.

[ref31] (2009). Hemostatic role of a folkloric medicinal plant extract in a rat partial nephrectomy model: controlled experimental trial. The Journal of urology.

[ref32] (2013). Medicinal plant extract (Ankaferd Blood Stopper) application in deep tissue injuries in rats: histopathological investigation of the effect on regional and systemic tissues. Ulus Travma Acil Cerrahi Dergisi.

[ref33] (2019). An immunohistochemistry and histopathological study of ankaferd blood stopper in a rat model of cervical inflammation. Revista da Associação Médica Brasileira.

[ref34] (2019). The Effectiveness of Ankaferd Blood Stopper in the Management of Traumatic Bleeding.

[ref35] (2018). Ankaferd blood stopper as a new strategy to avoid early complications after transradial procedures: A randomized clinical trial. Journal of interventional cardiology.

[ref36] (2012). Effects of folk medicinal plant extract Ankaferd Blood Stopper® in vital primary molar pulpotomy. European Archives of Paediatric Dentistry.

[ref37] (2011). The hemostatic efficacy of Ankaferd Blood Stopper in adenoidectomy. International journal of pediatric otorhinolaryngology.

[ref38] (2010). Prospective, randomized, controlled clinical trial of Ankaferd Blood Stopper in patients with acute anterior epistaxis. European Archives of Oto-Rhino-Laryngology.

[ref39] (2009). Prospective, controlled clinical trial of Ankaferd Blood Stopper in children undergoing tonsillectomy. International Journal of Pediatric Otorhinolaryngology.

[ref40] (2011). The efficacy of Ankaferd Blood Stopper for the management of bleeding following total thyroidectomy. Journal of Investigative Surgery.

[ref41] (2016). Ankaferd blood stopper enhances healing after osseous grafting in patients with intrabony periodontal defects. Journal of periodontal research.

[ref42] (2013). A new hemostatic agent (Ankaferd Blood Stopper®) in tubeless percutaneous nephrolithotomy: A prospective randomized study. Journal of endourology.

[ref43] (2014). Correlation between international normalized ratio values and sufficiency of two different local hemostatic measures in anticoagulated patients. European journal of dentistry.

[ref44] (2015). Local use of ankaferd blood clotter in emergent beating heart coronary artery bypass grafting. The open cardiovascular medicine journal.

[ref45] (2011). Haemostatic effect of Ankaferd Blood Stopper® seen during adenoidectomy. African Journal of Traditional, Complementary and Alternative Medicines.

[ref46] (2015). Ankaferd blood stopper decreases postoperative bleeding and number of transfusions in patients treated with clopidogrel: a double-blind, placebo-controlled, randomized clinical trial. in The heart surgery forum.

[ref47] (2013). Ankaferd blood stopper in episiotomy repair. Clinical and Experimental Obstetrics Gynecology.

[ref48] (2015). Safety and efficacy of Ankaferd hemostat (ABS) in the chemotherapy-induced oral mucositis. International Journal of Hematology and Oncology.

[ref49] (2009). Hemostasis in Retropubic Radical Prostatectomy with Ankaferd BloodStopper®:. A Case Report. The Kaohsiung journal of medical sciences.

[ref50] (2010). First clinical experience of Ankaferd BloodStopper as a hemostatic agent in partial nephrectomy. The Kaohsiung journal of medical sciences.

[ref51] (2010). Instant control of fundal variceal bleeding with a folkloric medicinal plant extract: Ankaferd Blood Stopper. Gastrointestinal endoscopy.

[ref52] (2010). Efficacy of Ankaferd Blood Stopper in postpolypectomy bleeding. The Journal of Alternative and Complementary Medicine.

[ref53] (2008). Successful management of bleeding due to solitary rectal ulcer via topical application of Ankaferd blood stopper. The Journal of Alternative and Complementary Medicine.

[ref54] (2010). The effect of a new hemostatic agent for difficult cases of non-variceal gastrointestinal bleeding: Ankaferd blood stopper.

[ref55] (2011). Upper gastrointestinal bleeding in a patient with defective hemostasis successfully treated with ankaferd blood stopper. Phytotherapy research.

[ref56] (2010). A new hemostatic agent—ankaferd blood stopper: management of gastrointestinal bleeding in an infant and other experiences in children. Pediatric hematology and oncology.

[ref57] (2010). A new practical alternative for the control of sternal bleeding during cardiac surgery: Ankaferd Blood Stopper. Heart Surgery Forum.

[ref58] (2012). Prohemostatic and antithrombin activities of Ankaferd hemostat are linked to fibrinogen gamma chain and prothrombin by functional proteomic analyses. Clinical and Applied Thrombosis/Hemostasis.

[ref59] (2018). Ankaferd Blood Stopper with antibiofilm potential successfully inhibits the extracellular matrix degradation enzymes and promotes wound healing of 3T3 fibroblasts in vitro. Turkish journal of medical sciences.

[ref60] (2017). A holistic anti-aging approach applied in selected cultivated medicinal plants: a view of photoprotection of the skin by different mechanisms. Industrial crops and products.

[ref61] (2016). Nettle (Urtica dioica L.) as a source of antioxidant and anti-aging phytochemicals for cosmetic applications. Comptes Rendus Chimie.

[ref62] (2018). Ankaferd blood stopper accelerates deep second degree burn wound healing in rats. Acta Veterinaria Brno.

[ref63] (2018). On Being a “Physician Patient” with His Own Experimental Therapeutic Drug. Turkish Journal of Hematology.

[ref64] (2015). et al. Indian Journal of Surgery.

[ref65] (2015). Severe radiation esophagitis successfully treated with Ankaferd hemostat. Gastrointestinal endoscopy.

[ref66] (2019). Therapeutic and preventative effects of ankaferd blood stopper in an experimental necrotizing enterocolitis model. Biomedicine & Pharmacotherapy.

[ref67] (2019). The efficacy of Ankaferd Blood Stopper® in an experimental Asherman syndrome model created in rats. Turkish journal of obstetrics and gynecology.

[ref68] (2016). In vitro extraction and fermentation of polyphenols from grape seeds (Vitis vinifera) by human intestinal microbiota. Food & function.

[ref69] (2009). Antimicrobial activity of plant extract Ankaferd Blood Stopper®. Fitoterapia.

[ref70] (2008). In vitro anti-bacterial activities of Ankaferd blood stopper. International Journal of Laboratory Hematology.

[ref71] (2009). M Turgut et al. Turkiye Klinikleri Tip Bilimleri Dergisi.

[ref72] (2011). In vitro antifungal activity of Ankaferd Blood Stopper against Candida albicans. Current Therapeutic Research.

[ref73] (2019). Effects of Ankaferd Hemostat on Helicobacter pylori strains and antibiotic resistance. Turkish journal of medical sciences.

[ref74] (2013). In vitro effect of Ankaferd Blood Stopper®, a plant extract against Mycobacterium tuberculosis isolates. Mikrobiyoloji bulteni.

[ref75] (2016). Sipahi M et al. Iranian journal of parasitology.

[ref76] (2018). Effectiveness of Ankaferd BloodStopper in Prophylaxis and Treatment of Oral Mucositis in Childhood Cancers Evaluated with Plasma Citrulline Levels. Turkish Journal of Hematology.

[ref77] (2016). Qualitative/chemical analyses of Ankaferd hemostat and its antioxidant content in synthetic gastric fluids. BioMed research international.

[ref78] (2014). Evaluation of anti-neoplastic effects of a new hemostatic agent Ankaferd blood stopper on myeloma cell line and plasmocytoma development in Balb/c mice: results of the first in vitro and in vivo study. Blood.

[ref79] (2018). Evaluation of the chemopreventive effects of ankaferd bloodstopper in 7, 12-dimethylbenz [a] anthracene-induced oral epithelial dysplasia. Clinical oral investigations.

[ref80] (2013). Short-term effects of Ankaferd hemostat for renal artery embolization: an experimental study. Cardiovascular and interventional radiology.

[ref81] (2016). Splenic artery embolization with Ankaferd blood stopper in a sheep model. Diagnostic and Interventional Radiology.

[ref82] (2011). Percutaneous ankaferd injection to in vivo liver tissue in comparison to ethanol in an experimental rat model. Clinics and research in hepatology and gastroenterology.

